# On Calcareous Metastases

**Published:** 1855-09

**Authors:** Rudolph Virchow


*On Calcareous Metastases.* By RUDOLPH VIRCHOW.—Some years ago I was called on, in Berlin, to make the examination of the body of a young lady, the course of whose disease had baffled the diagnostic and therapeutic skill of her attendants. A member of one of the highest families, she had, in the bloom of youth and beauty, and without any assignable cause, begun to complain of dragging pains in the most different parts of the body; in spite of the most attentive treatment by physicians of the greatest experience, this apparent rheumatism steadily increased, the nutrition of the body was finally interfered with, and death put an end to her severe suffering, without any evident local affection having been discovered. The autopsy exhibited very numerous and extensive cancerous nodules in almost all the larger bones of the skeleton, particularly in the bodies of the vertebræ and the bones of the skull. But nowhere did these nodules project above the osseous surface, they were in almost every situation covered with periosteum without any material alteration of the level, and lay in large, irregularly corroded chasms in the bones. The loss which the latter suffered in their constituent elements, and especially in their calcareous parenchyma, was consequently very considerable.

Accordingly, it did not appear strange when, on examining the kidneys, we found in the pelvis and calyces great quantities of a dirty white deposit, in some parts sandy, in others crumbling, consisting principally of earthy carbonates and phosphates. But I was not a little surprised, on cutting through the lungs, to find in their central portions singularly dry patches of considerable extent, of a grayish white color, and rough to the touch, permeable to the air, but offering considerable resistance to the knife; and likewise to encounter in the stomach very extensive whitish parts, also very dry, and feeling almost sandy: a closer examination of both of which showed that the parenchyma of the lungs, and the mucous membrane of the stomach, were throughout overloaded with calcareous salts.

I had never before seen these changes, but they were so well marked that it appeared scarcely possible to doubt that we had to do with a metastatic deposit of those salts of lime which had been removed by absorption from the cavities in the bones. In rickets it has, indeed, been known since the celebrated case of Madame Supiot, that the urine in the commencement of the disease lets fall a chalky deposit, and nothing is more expressive than the remark of this unfortunate patient, that her limbs "were fermenting," when her urine was loaded with a large quantity of the sediment. Must we not in this case infer that the earthy salts which pass off with the urine are really derived from the bones?

If we add the experience afforded by the study of gout, where the urates which should be excreted by the kidneys become deposited on the joints, and, indeed, as Schroeder Van der Kolk has shown, on the tendons, ligaments, skin, nerves, and vessels, we shall be led to conclude that the salts of lime which in cases of great destruction to the bones, reach the blood in increased quantity, are first of all separated through the kidneys, but that when this mode of elimination is insufficient, other parts of the body become filled with them. Thus, also, we observe that jaundice does not become very evident until the elimination of the coloring matter of the bile by the urine is not sufficient to purify the blood, and if time should hereafter confirm the observation of H. Meckel, who found the Malphigian tufts full of salts of silver, in an epileptic person long treated with the nitrate of its oxide,—the well-known coloring of the external parts after the use of lunar caustic would certainly admit of our inferring the existence of a similar condition.

The observations which have been made in reference to the retention of urea, particularly those of Bernard, become of especial interest in connection with this subject. In such cases it would appear that the urea accumulated in the blood also effects its metastases, and that chiefly in the stomach abundant elimination of carbonate of ammonia takes place, while the strongly ammoniacal smell evolved from the lungs of uremic patients has long been familiar to the physician.

Little has been said by writers upon the calcification of the lungs and stomach. Voigtel has collected a great number of observations on ossification of these organs, yet none of them appear to correspond exactly to the case before us. The same may be said of the quotations of Cruveilhier, Joh. Fr. Meckel, and Hodgkin, for in this case we have neither a formation of bone, nor a peculiar concretion, but a fine infiltration or incrustation of the parts. In more recent authors I find nothing at all upon this point.—*Dublin Quarterly Journal.*

